# Characteristics of Severe Retinopathy of Prematurity in Infants with Birth Weight above 1500 Grams at a Referral Center in Turkey

**DOI:** 10.1371/journal.pone.0161692

**Published:** 2016-08-22

**Authors:** Murat Gunay, Gokhan Celik, Abdulhamit Tuten, Guner Karatekin, Handan Bardak, Fahri Ovali

**Affiliations:** 1 Department of Ophthalmology, Zeynep Kamil Maternity and Children’s Diseases Training and Research Hospital, Istanbul, Turkey; 2 Department of Neonatology, Zeynep Kamil Maternity and Children’s Diseases Training and Research Hospital, Istanbul, Turkey; 3 Department of Ophthalmology, Haydarpasa Numune Training and Research Hospital, Istanbul, Turkey; 4 Department of Pediatrics, Division of Neonatology, Istanbul Medeniyet University, Faculty of Medicine, Istanbul, Turkey; Justus Liebig Universitat Giessen, GERMANY

## Abstract

**Purpose:**

To demonstrate the clinical characteristics and treatment outcomes of severe retinopathy of prematurity (ROP) in preterm infants with birth weight (BW) above 1500 g in Turkey.

**Methods:**

A retrospective review of 5920 ROP records was performed in Zeynep Kamil Maternity and Children’s Diseases Training and Research Hospital. The records were obtained from ROP treatment center of the same institute between 2011 and 2016. The data comprised the demographic and clinical characteristics including, gestational age, BW, systemic risk factors, zone and stage of ROP, ROP type, treatment modality, treatment outcomes and inborn/outborn status of the babies.

**Results:**

A total of 36 infants (71 eyes) with severe ROP and BW> 1500 g were retrieved. There were 30 infants (83.3%) with type 1 ROP and 6 infants (16.7%) with aggressive posterior ROP (APROP). 3 infants (8.3%) were born at our hospital whereas 33 (91.7%) were referred from outer private neonatal intensive care unit (NICU) centers. Zone I APROP was detected during the initial screening. 21 infants (58.3%) underwent laser treatment while 15 (41.7%) received intravitreal bevacizumab (IVB) injections. No unfavorable structural outcome was observed following either treatment modality.

**Conclusion:**

Severe ROP may occur in heavier preterm infants. Laser treatment and IVB injections were useful in selected cases. Presence of APROP at first examination suggests an earlier screening in heavier babies. Standardization of private NICU centers as well as establishing a national ROP protocol is necessary in Turkey.

## Introduction

Retinopathy of prematurity (ROP) is a leading cause of treatable childhood blindness which is characterized by abnormal retinal vascular development [1]. It may progress to retinal detachment if more severe disease is left untreated. An early and appropriate treatment plays a great role in infants who are at risk for progression to retinal detachment [2]. Lower gestational age (GA) and birth weight (BW) have been shown as the main risk factors for the development of ROP [3]. However, studies from developing and middle-income countries have indicated that more mature preterm babies can develop severe ROP [4]. It has been stated that Western ROP screening guidelines [5,6] might not be appropriate to detect all infants at risk in these regions resulting in a significant number of infants are being missed who can require treatment [7–9]. Furthermore, in Turkey, several reports on ROP screening included neonates with GA ranged from 32 weeks to 37 weeks or BW between 1500 g and 2000 g, and specified that more mature babies can develop severe ROP [10–13]. However, data regarding the clinical characteristics and treatment outcomes of severe ROP in heavier preterm infants are limited in Turkey.

In the present study, our aim was to document the characteristics and outcomes of heavier premature infants treated at our referral center between the period 2011 to 2015.

## Materials and Methods

The study was approved by the Institutional Review Board (IRB) of Zeynep Kamil Maternity and Children’s Diseases Training and Research Hospital and conducted according to the principles laid out in the Declaration of Helsinki. A retrospective chart review of ROP records from 2011 to 2015 was performed. Patient records and information were anonymized and de-identified prior to analysis. Examinations were performed on infants born with GA≤32 weeks and BW≤1500 g as well as those with GA>32 weeks and BW>1500 g who were believed by their pediatrician to be at high risk for development of ROP. Infants with BW above 1500 g who were treated for ROP were retrieved for the study. The patient demographics including gender, GA and BW, zone and stage of ROP, type of ROP (Type 1 ROP or aggressive posterior ROP (APROP)), treatment time, treatment modality including laser photocoagulation (LPC) or intravitreal bevacizumab (IVB) injection, follow-up time after treatment, anatomic outcomes with refractive data at last visit were recorded. Systemic risk factors that would have a possible association with the development of ROP were also noted.

### Screening and Treatment Procedures

Infants were first screened at postnatal 4 weeks. Treatment was considered in case of the development of type 1 ROP and APROP. Type 1 ROP was defined as stated in the current guidelines as, Zone I any stage ROP with plus disease, Zone I stage 3 ROP with or without plus disease, Zone II stage 2 or 3 ROP with plus disease [14]. APROP was defined as stated in International Classification of ROP (ICROP) study which includes, in Zone I or posterior Zone II, increased dilation and tortuosity of the posterior pole vessels in all quadrants out of proportion to the peripheral retinopathy with flat network of shunts within retina or at the junction between vascular and avascular retina [15].

The LPC or IVB (Altuzan, 100 mg/4 ml flacon, Roche, Turkey) were performed for the treatment of ROP. Indications for treatment were determined based upon the criteria suggested by the Early Treatment for ROP (ETROP) study [14]. Informed consent was obtained from all parents before the treatments. Infants who had ROP in Zone I or posterior Zone II, injections of IVB were administered based upon the results provided by Bevacizumab Eleminates the Angiogenic Threat of ROP (BEAT-ROP) study [16]. Other infants in the study underwent laser treatment.

Laser treatment was performed under topical anesthesia with a 810 nm diode laser device (Iridex; Oculight SL, Mountainview, CA, U.S.A). Follow-up examinations were continued 1 to 3 weeks or monthly intervals until complete regression of the disease was achieved. The IVB injections were performed after providing aseptic conditions. The standard injection technique was applied similar to that stated in previous literature [17]. Continued examinations were performed weekly or bi-weekly for the first month, every three weeks for two additional months and then monthly until vascularization of the peripheral retina was achieved in Zone III without any active component including tractional tissues or retinal detachment.

Retinal images were obtained with wide-field digital imaging system using RetCam Shuttle device (Clarity Medical Systems Inc., Pleasanton, CA, USA).

### Evaluation of Treatment Outcomes

At the end of the follow-up, unfavorable anatomic outcome was considered if one of the following situations were observed; dragging of the disc, localized tractional or nontractional membranes at posterior pole or in the retinal periphery, total or partial retinal detachment, those of which were identical to the outcomes stated in the previous literature [14].

Refractive data were obtained at last visit from each child. Refractive errors were assessed 45 minutes after three instillations of cyclopentolate 1% at 10-minute intervals by using retinoscopy.

## Results

A total of 5920 infants were screened for ROP in Zeynep Kamil Maternity and Children’s Disease Training and Research Hospital between the years 2011 and 2015. Of these infants, 36 (0.61%) with BW >1500 g developed ROP requiring treatment. Among the screened infants, a total of 2100 infants with BW>1500 g received ROP screening during the study period at our center. There were 19 males (52.8%) and 17 females (47.2%). The complete data regarding the clinical and treatment characteristics of the study population are summarized in Table 1. The largest baby in this study was a male infant with GA of 36 weeks and BW of 2900 g (patient no:21).

**Table 1:** Demographic data and clinical characteristics of the infants.

Patient No	Gender	GA(weeks)	BW(g)	Zone	ROPType	ROP Stage in Type 1 ROP	Treatment time(PMA,weeks)	Treatment	Follow-up(months)
**1**	Male	31	1520	OU: II	Type 1	3	36	LPC	47
**2**	Male	31	1520	OD: II	Type 1	3	39	LPC	16
**3**	Male	31	1600	OD: II	Type 1	3	40	LPC	21
**4**	Male	31	1610	OU: II	Type 1	3	37	LPC	8
**5**	Female	32	1650	OU: II	Type 1	3	38	LPC	10
**6**	Male	32	1650	OU: II	Type 1	3	37	LPC	32
**7**	Female	31	1660	OU: II	Type 1	3	36	LPC	11
**8**	Male	33	1700	OU: II	Type 1	3	40	LPC	10
**9**	Male	32	1700	OU: II	Type 1	3	38	LPC	45
**10**	Male	31	1700	OU: II	Type 1	3	37	LPC	13
**11**	Female	31	1730	OU: II	Type 1	3	38	LPC	8
**12**	Female	32	1800	OU: II	Type 1	3	37	LPC	9
**13**	Male	32	1800	OU: II	Type 1	3	39	LPC	46
**14**	Male	33	1810	OU: II	Type 1	3	40	LPC	43
**15**	Male	32	1840	OU: II	Type 1	3	38	LPC	8
**16**	Female	32	1900	OD: II	Type 1	3	38	LPC	18
**17**	Female	33	1910	OU: II	Type 1	3	38	LPC	8
**18**	Female	32	2000	OU: II	Type 1	3	38	LPC	21
**19**	Female	32	2060	OD: II	Type 1	3	36	LPC	10
**20**	Male	32	2100	OU: II	Type 1	3	36	LPC	21
**21**	Male	36	2900	OU: II	Type 1	3	42	LPC	12
**22**	Male	34	1510	OU: II	Type 1	3	40	IVB	12
**23**	Female	32	1530	OU: I	APROP		36	IVB	26
**24**	Male	31	1540	OU: I	APROP		35	IVB	26
**25**	Male	32	1550	OU: I	APROP		36	IVB	11
**26**	Male	31	1570	OU: II	Type 1	3	37	IVB	27
**27**	Female	32	1645	OU: I	APROP		36	IVB	23
**28**	Male	33	1770	OU: II	Type 1	3	37	IVB	12
**29**	Male	31	1800	OU: I	APROP		35	IVB	11
**30**	Male	31	1810	OU: II	Type 1	3	35	IVB	33
**31**	Female	30	1875	OU: II	Type 1	3	36	IVB	13
**32**	Female	32	1940	OU: II	Type 1	3	36	IVB	13
**33**	Male	35	2145	OU: II	Type 1	3	42	IVB	12
**34**	Female	34	2370	OS: II	Type 1	3	38	IVB	14
**35**	Female	32	2710	OU: I	APROP		36	IVB	22
**36**	Male	34	2900	OU: II	Type 1	3	39	IVB	14

GA: gestational age; BW: birth weight; PMA: postmenstrual age; LPC: laser photocoagulation; IVB: intravitreal bevacizumab

Patients are ordered by birth weight within LPC and IVB groups.

Table 2 shows the demographic and refractive data of the treated infants in the study.

**Table 2:** Demographic data and refractive outcomes of laser and IVB treated infants.

	LPC group(N = 21)	IVB group(N = 15)	p^a^
GA, mean, weeks	32.00	32.27	0.61
BW, mean, g	1816.19	1907.67	0.94
PMA at treatment, mean, weeks	38.00	36.93	*0*.03*
Follow-up, mean,months	19.86	17.93	0.37
SE, mean, diopters	0.17	-0.83	0.98

^a^Mann-Whitney U Test

p<0.05*

LPC: laser photocoagulation; IVB: intravitreal bevacizumab; GA: gestational age; BW: birth weight; PMA: postmenstrual age; SE: spherical equivalent; CI: confidence interval

Three infants (8.3%) were born at our hospital whereas 33 (91.7%) were referred from outer private neonatal intensive care unit (NICU) centers. Thirty infants with type 1 ROP (83.3%) had Zone II stage 3 ROP with plus disease and 6 (16.7%) had the diagnosis of Zone I APROP. One infant (case no:30) who was diagnosed with Bartter syndrome at our hospital showed Zone II posterior type 1 ROP. Infants with Zone I APROP were diagnosed during the initial screening examinations in the study. All infants with APROP were born at outer private NICUs.

**Fig 1:**
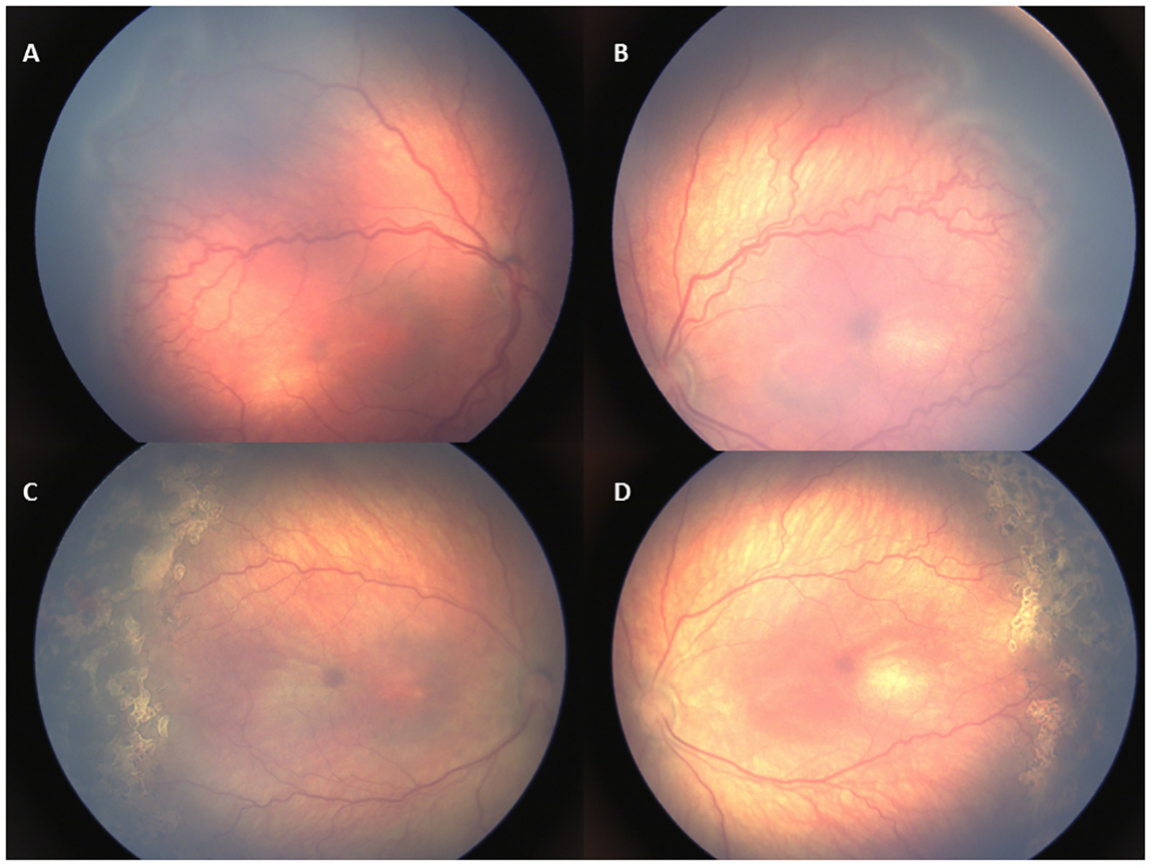
Retinal images of an infant with type 1 ROP (case no:8). (A,B) Zone II stage 3 ROP with plus disease before treatment. (C,D) Plus disease and ROP regressed 1 week following laser treatment.

**Fig 2:**
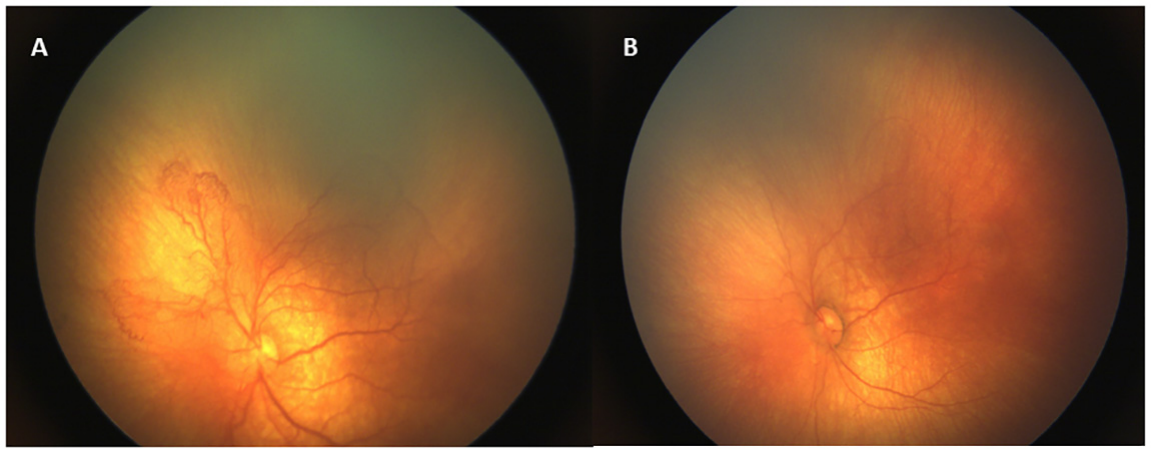
Retinal images of an infant with APROP (case no:29). (A) Intensive shunt vessels with plus disease indicate Zone I APROP. (B) A dramatic response to IVB injection with regression of plus disease and resolution of shunt vessels 1 week following treatment.

Laser treatment was performed in 21 infants (58.3%), while 15 (41.7%) received IVB injections. All infants who underwent laser ablation had anterior Zone II stage 3 ROP with plus disease (Fig 1). Regarding IVB treatment, 6 infants (40%) had Zone I APROP (Fig 2), 9 infants (60%) had posterior Zone II type 1 ROP with plus disease.

Risk factors of the study population are summarized in Table 3. The mean period of oxygen administration was 11.1±4.7 days (5 to 26 days).

**Table 3:** Risk factors of the study population (N = 36).

Risk Factors	N (%)
C/S	28 (77.8%)
Twin delivery	4 (11.1%)
RDS	19 (52.3%)
Sepsis	13 (36.1%)
Pneumonia	14 (38.9%)
BPD	3 (8.3%)
Neonatal jaundice	17 (47.2%)
Bartter syndrome	1 (2.8%)

C/S: cesarean section; RDS: respiratory distress syndrome; BPD: bronchopulmonary dysplasia

Any patient may have more than one risk factor.

No unfavorable outcome was observed in infants treated either with LPC or IVB at the end of the study. No complication was noted related to the treatment procedures. All eyes who received IVB showed peripheral retinal vascularization in Zone III. No additional treatment was performed following either LPC or IVB.

## Discussion

The present study demonstrated that severe ROP is still a problem among more mature preterm neonates in Turkey. We found an incidence 0.61% of severe ROP in infants with BW>1500 g. Although most of the study population were diagnosed with type 1 ROP, 6/36 of the infants showed Zone I APROP. Laser treatment and IVB injection seemed to be effective in selected cases in our study.

Several studies from low and middle-income countries previously drew attention to severe ROP in larger premature infants. It has been shown that presence of APROP is common in larger premature infants [7–9,18]. Shan et al. [19] recently reported type 1 ROP in infants with BW >1250 g in China. Shah et al. [20] observed Zone I APROP in premature infants with higher GA and BW and stated that outborn status with uncontrolled oxygen administration were the main factors contributing development of APROP. We found an incidence 0.51% of type 1 ROP and 0.11% of APROP in the present study.

Besides lower GA and BW, presence of several comorbidities in preterm neonates including, patent ductus arteriosus, sepsis, intraventricular hemorrhage (IVH), ventilator dependence, thrombocytopenia, anemia, necrotizing enterocolitis, bronchopulmonary dysplasia (BPD) and respiratory distress syndrome (RDS) contribute development of severe ROP [21,22]. Chen et al. [23] found that infants with higher BW who have an IVH tend to develop severe ROP. Hungi et al. [9] identified RDS, sepsis, neonatal jaundice and oxygen therapy as risk factors for the development of severe ROP in greater preterms. We observed 19 infants (52.3%) with RDS, 13 (36.1%) with sepsis, 14 (38.9%) with pneumonia, 3 (8.3%) with BPD and 17 (47.2%) with neonatal jaundice. Furthermore, one interesting observation in an infant with 31 weeks of gestation and 1810 g of BW, was a diagnosis of Bartter syndrome in whom we detected posterior Zone II ROP with plus disease at first screeening examination. Bartter syndrome is characterized by hypokalaemic, hypochloraemic metabolic alkalosis associated with increased urinary loss of sodium, potassium, calcium and chloride [24]. Patients with Bartter syndrome have hyperreninaemia and hyperaldosteronaemia as well as increased angiotensin II activity [25]. It has been shown that an interaction between the angiotensin II receptor and vascular endothelial growth factor (VEGF) exists in retinal angiogenesis process [26]. To the best of our knowledge, this is the first report of severe ROP in a heavier premature neonate with Bartter syndrome. In our opinion, this intriguing finding needs to be further investigated in consideration of the relationship between angiotensin II and VEGF.

Most of the studies have emphasized the improper unblended oxygen administration as well as insufficient neonatal care in outer centers as essential risk factors for the development of severe ROP in infants with higher GA and BW [9,20, 27,28]. Although we noted several risk factors, these were not sufficient to explain the development of severe ROP in larger premature infants in our study. We were not able to reach the detailed patient records including their oxygen concentration, but we noted a relatively lower duration of oxygen supplementation among these infants. Importantly, we realized that no on-site neonatologist exists at outer private NICUs included in our study and infants were managed by general pediatricians in these units. It would be reasonable that excessive and inappropriate oxygen therapy with inadequate care might lead to the clinical picture seen in these babies. Supportively, Bas et al. [29] studied the incidence and severity of ROP by including data from 49 NICUs in Turkey and revealed a significantly higher incidence of severe ROP at outer private hospital NICUs than the university and state hospital NICUs. Here we suggest that lack of neonatologists as well as insufficient nursing care in outer NICU centers seemed to be an important problem to obtain an adequate neonatal care in Turkey.

A recent report in Turkey indicated that infants with GA and BW under or equal to 33 weeks and 1770 g should be screened in Eastern Black Sea region of Turkey [30]. Another study reported that infants with GA and BW above 32 weeks and 1500 g developed ROP in Turkey [31]. The largest infants in our study had GA ranged from 34 to 36 weeks with BW above 1500 g. Therefore BW may principally be considered to establish screening criteria in Turkey rather than GA. Most importantly, we also detected Zone I APROP during the initial screening examinations. Based on this finding, for the first time we may suggest that heavier preterm infants in Turkey should be examined earlier irrespective of GA, at 2 or 3 weeks after birth as indicated in other studies from Asian region [32,33].

The major drawback of the present study was its retrospective design including observation of the data from a single center, thereby, interpretation of our findings should be made carefully. However, as our institute is one of the largest referral center for ROP treatment in Turkey, our results are still noteworthy by revealing the clinical characteristics of severe ROP in preterm babies with BW above 1500 g. Actually, no current consensus on national ROP screening strategy is present in Turkey. It is clearly seen that an elaborative national screening guideline in Turkey needs to be established for ROP. Additionally, it seems more logical to consider BW rather than GA, to initiate screening in heavier preterm babies.

In conclusion, infants with BW above 1500 g still develop severe ROP in Turkey. Based on the data provided from our study, we may offer an initial screening earlier than postnatal 4 weeks in heavier neonates in order to timely detect severe ROP. Moreover, in view of the higher percentage of outborn status of the infants in the present study, standardization of outer private NICU centers would be reasonable in Turkey in terms of better administration of oxygen therapy with sufficient neonatal care.
